# Convenience stores: an obesogenic promoter in a metropolitan area of northern Mexico?

**DOI:** 10.3389/fnut.2024.1331990

**Published:** 2024-03-06

**Authors:** Marco Antonio Ávila Arcos, Teresa Shamah Levy, Marti Yareli Del Monte Vega, Adolfo Chávez Villasana, Abelardo Ávila Curiel

**Affiliations:** ^1^Center for Research on Evaluation and Surveys, National Institute of Public Health of Mexico, Cuernavaca, Mexico; ^2^Applied Nutrition and Nutritional Education Department, National Institute of Medical Sciences and Nutrition Salvador Zubirán, Mexico City, Mexico

**Keywords:** childhood obesity, geographic information systems, obesogenic environment, spatial analisis, scholar population

## Abstract

**Introduction:**

The prevalence of obesity in the Mexican school-age (5–11 years old) population increased from 8.9 to 18.1% between 1999 and 2022. Although overweight and obesity (OW + Ob) is a complex and multifactorial phenomenon, alongside its increasing trend, changes in eating patterns as a result of obesogenic environments that promote higher energy intake have been documented. The objective of the present study was to detect possible associations between schools and their proximity to and density of convenience stores in Monterrey, Mexico from 2015 to 2018.

**Materials and methods:**

Anthropometric data were obtained from a subset of measurements of the National Registry of Weight and Height (RNPT) performed in the Monterrey Mexico metropolitan area in 2015 and 2018, and obesity prevalence was computed and classified into quintiles at the school level. Convenience store data were obtained from the National Directory of Economic Units (DNUE). The analyses consisted of densities within 400-800 m buffers, distance to the nearest stores, and cartographic visualization of the store’s kernel density versus OW + Ob hotspots for both periods.

**Results:**

A total of 175,804 children in 2015 and 175,964 in 2018 belonging to 1,552 elementary schools were included in the study; during this period, OW + Ob prevalence increased from 38.7 to 39.3%, and a directly proportional relationship was found between the quintiles with the higher OW + Ob prevalence and the number of stores for both radii. Hotspots of OW + Ob ranged from 63 to 91 between 2015 and 2018, and it was visually confirmed that such spots were associated with areas with a higher density of convenience stores regardless of socioeconomic conditions.

**Conclusion:**

Although some relationships between the store’s proximity/density and OW + Ob could be identified, more research is needed to gather evidence about this. However, due to the trends and the magnitude of the problem, guidelines aimed at limiting or reducing the availability of junk food and sweetened beverages on the school’s periphery must be implemented to control the obesogenic environment.

## 1 Introduction

Overweight and Obesity (OW + Ob) is among the most challenging and urgent problems worldwide. In the last decade, no countries have controlled or even lowered their OW + Ob prevalence, particularly in countries with lower incomes, which report greater increases in this issue (World Obesity Federation Atlas) for the period between 2020 and 2025 on a worldwide scale. An increment is expected in the prevalence of obesity from 10 to 14% and 8 to 10% for boys and girls aged 5–19 years, respectively (1).

In Mexico, the OW + Ob problem was recognized in 2016 and ratified in 2018 as a national public health emergency (2). In the school-age population (5–11 years old), the National Nutrition Survey (ENSANUT) reported an alarming increase between 1999 and 2022, rising from 17.2 to 19.2% for overweight and from 8.3 to 18.1% for obesity (3, 4).

This rising trend in OW + Ob has occurred alongside changes in eating patterns, and Popkin et al. found a shift from home-prepared to processed and packaged foods (5). This is not unique to Mexico; in other countries such as Brazil, the increase in ultra-processed foods (junk food) during the last two decades has also been documented (6).

There is existing literature that demonstrates a link between junk food consumption and OW + Ob as a “cause-effect” relationship. One such study was developed by PAHO in 2015, wherein the authors analyzed data from 14 countries and found a significant relationship between the per-capita sales of these products and the OW + Ob levels. Moreover, in their conclusion, they encourage countries to reduce the consumption of ultra-processed food due to its negative impacts on the nutrition of the population (7). Longitudinal (cohort) studies (8) and literature reviews (9) have confirmed this relationship. Recently, a study conducted in eight countries (including Mexico) documented that increased consumption of ultra-processed foods was associated with higher energy and free sugar intake and stated that this association constituted a potential determinant of obesity in children and adolescents (10).

Numerous studies have been conducted to examine the obesogenic environment from a spatial perspective, some of them in Latin American countries such as Peru and El Salvador (11, 12), which are merely descriptive and aimed at characterizing the spatial distribution of OW + Ob. In the case of seeking spatial interactions between junk food offerings and OW + Ob, research is mainly based on developed countries, and one study conducted in the United Kingdom in 2012 established that areas with greater access to fast food stores also had greater OW + Ob prevalence (13). Similar studies have been carried out in the USA, Netherlands, Germany, Canada, Macao, and New Zealand, finding spatial interactions between food sources and OW + Ob (14–18).

In Mexico, recent studies have approached this issue by conducting a cross-sectional analysis to estimate the indirect association between food store density and OW + Ob among Mexican adolescents, using sugar-sweetened beverage (SSB) consumption as a mediator. Store density was directly associated with SSB consumption but not indirectly associated with OW + Ob mediated by SSB (19). Another study analyzed the change in the retail food environment in Mexican municipalities from 2010 to 2020 and assessed whether these trends were modified by socioeconomic deprivation. It was concluded that there has been a substantial expansion and rapid change in Mexico’s food environment, driven mainly by the rise in convenience stores and supermarkets in the most deprived and least urbanized areas (20).

The present study was designed to measure the impact of convenience stores on OW + Ob prevalence in children, which is relevant because some authors have documented that although the home is still a place where a large number of calories is consumed, a significant proportion of the calories consumed (almost one-third) come from eating episodes conducted outside of the house (21, 22). A spatial approach using GIS techniques was proposed, considering that the periphery of schools could be a place where children acquire a significant proportion of their calories.

The objective of the present study was to assess the OW + Ob rates in children who attended schools in Monterrey, Mexico, between 2015 and 2018, and to detect possible associations between schools and their proximity to and density of convenience stores.

## 2 Materials and methods

The present work is an ecological study whose population was children (6 to 12 years old) from Monterrey, Mexico; the variable of interest was OW + Ob prevalence at the school level and its relation to spatial attributes – proximity and density – of convenience stores, as promoters of obesogenic environments. Considering the school as the unit of analysis, the study is longitudinal (2015–2018) since anthropometric data are present in both periods for the 1,552 schools.

### 2.1 Data sources

The study area encompasses the Monterrey Metropolitan Area, situated in the northern region of Mexico (25.67° N, 100.308° E), approximately 224 km southwest of the United States border in Laredo, Texas. As of the 2020 census, this region comprises 16 municipalities, analogous to county-level administrative units, with a total population of 5.3 million inhabitants. The economic landscape of the area is predominantly shaped by industrial activities, with a particular emphasis on key sectors such as beer, steel, and concrete production and the manufacture of cars and machinery.

Anthropometric data were obtained from the National Registry of Weight and Height (RNPT in Spanish) (23), a strategy implemented in Mexico to evaluate the nutritional status of children in elementary schools. Its main objective is to identify nutritional disorders such as malnutrition, overweight, and obesity. This initiative is jointly managed by the Health and Education Ministries (SSA and SEP in Spanish) and the National and State Systems for Integral Family Development (DIF) and is technically overseen by the National Nutrition and Medical Sciences Institute (INCMNSZ). This collaboration includes periodic visits to schools by trained personnel to conduct anthropometric measurements (24, 25). From these data, a subset consisting of 1,552 Elementary schools belonging to the urban areas of the municipalities (a county-like territorial division) conforms to the Metropolitan Area of Monterrey, as defined by the National Statistics Geography and Informatics Institute (INEGI) in its Metropolitan Areas Catalog (26). This dataset had an OW + Ob prevalence for each school of 454,217 and 447,792 children in 2015 and 2018, respectively. Nutritional status was defined using the WHO BMI/age indicator, defining overweight as individuals with a z-score greater than +1 S.D. and obesity as those with a z-score > +2 S. D (27). To include socioeconomic data on the schools, a social exclusion index (SEI) variable built for the 2020 National Population and Household Census (28) was included, and the smallest geographic unit (suburb) was used to obtain the maximal resolution for characterizing the schools.

Convenience store data were extracted from the National Directory of Economic Units (DNUE) of INEGI (29), which includes information regarding all the businesses across the country, such as the type of activity, company name, number of employees, opening date, and, most important for the purposes of the present study, latitude and longitude. The final store dataset was constructed by filtering the DNUE database for the following criteria: “Convenience stores” as economic activity code, “OXXO” or “7-11” as company name, and – as in the case of the schools – being located in the urban areas of the Metropolitan Area of Monterrey. In 2015 and 2018, 1,394 and 1979 convenience stores were located, respectively (585 new stores were opened in the metropolitan area during this period).

### 2.2 Spatial and statistical analyses

All previously described data were clipped to the suburb polygon layer that included the SEI; quintiles for OW + Ob and the index were recoded to create categorical variables using QGIS 3.282 and SPSS 25, respectively (30, 31). Buffers (influence areas) of 400 and 800 m were constructed around every school using QGIS; these distances represent, in round numbers, walking times of 5 and 10 min, respectively, for an average pedestrian speed of 1.25 m/s (32).

As the geographic layers were not locally projected, we used the Python equidistance buffer plugin to generate buffer polygons with a precision of ±2 m (33). Once the polygons were generated, we used the QGIS *count points in the polygon* geoprocessing tool to determine the number of convenience stores within 400 m and 800 m radii from each school, which allowed us to estimate the density of such stores around the school-age population. Additionally, to establish ease of access to the stores from the schools, we computed the Euclidean distance from every school to its nearest store using the *distance to the nearest hub* geoprocessing tool. Once the spatial variables were calculated, cross-tabulations and scatter plots were performed to analyze the interaction between the density and ease of access of the population to stores with OW + Ob prevalence.

Additionally, to evaluate the impact of SEI characteristics on OW + Ob, a bootstrap analysis of 1,000 samples was performed to calculate the 95% confidence intervals for OW + Ob prevalence for every SEI quintile and determine whether the differences could be due to this and not necessarily because of the proximity and density of the convenience stores. Bootstrap analysis was performed using the SPSS v. 25.0, which uses the computer’s calculation power to create a large number (1,000 in this case) of subsamples of the actual data to calculate a standard error, thus obtaining a confidence interval of the variable of interest (OW + Ob prevalence) (34).

Finally, the last analysis consisted of generating a raster Kernel Density Estimation (KDE) heatmap for the stores in 2015 and 2018, using the QGIS *KDE* geoprocessing tool with an Epanechnikov kernel shape, which is the most efficient and suitable for our data (35), and a bandwidth of 0.24° (degrees), defined by Scott’s Rule for Bandwidth Selection (36) calculated for 2015 store data. The rasters corresponding to the density of the stores for the 2 years were clipped to the metropolitan area of Monterrey and cartographically represented with a pseudo-band color ramp for their visualization. Along with the previously described process, a *Getis*-Ord Gi* hotspot analysis was performed in ArcGIS desktop 3.0.3 (37) with the OW + Ob prevalence as the input field. This spatial statistical procedure allowed us to identify, with a significance level (90, 95, and 99%), those schools with a prevalence remarkably greater than their neighbors (38), and the resulting layer of this process was also included in the map along with the store density to visualize the relationship between them. This was performed for 2015 and 2018; therefore, changes over time can be observed when comparing the two maps.

## 3 Results

### 3.1 Overweight and obesity behaviors in schools

The number of children suffering from OW + Ob in the weight and height registry was 175,804 and 175,964 in 2015 and 2018, respectively, which are expressed as prevalence corresponding to 38.7 and 39.3%, respectively, which also modifies the Q4 and Q5 quintiles by year. The results are presented in Table 1.

**Table 1:** General characteristics of the elementary schools, Monterrey Metropolitan Area Mexico 2015–2018.

	2015	2018
Number of Schools	1,552	1,552
N total Children	454,218	447,792
N OW + Ob	175,804	175,964
Prevalence OW + Ob	38.70	39.30
OW + Ob Quintiles, N (interval)		
Q1	*n* = 310 (< 32.3)	*n* = 310 (<32.3)
Q2	*n* = 310 (32.3, 36.9)	*n* = 311 (32.3, 36.9)
Q3	*n* = 311 (36.9, 40.6)	*n* = 310 (36.9, 41.0)
Q4	*n* = 311 (40.6, 44.8)	*n* = 311 (41.0, 46.5)
Q5	*n* = 310 (>44.8)	*n* = 310 (>46.5)

### 3.2 Social exclusion index and its impact on OW + OB

**Figure 1:**
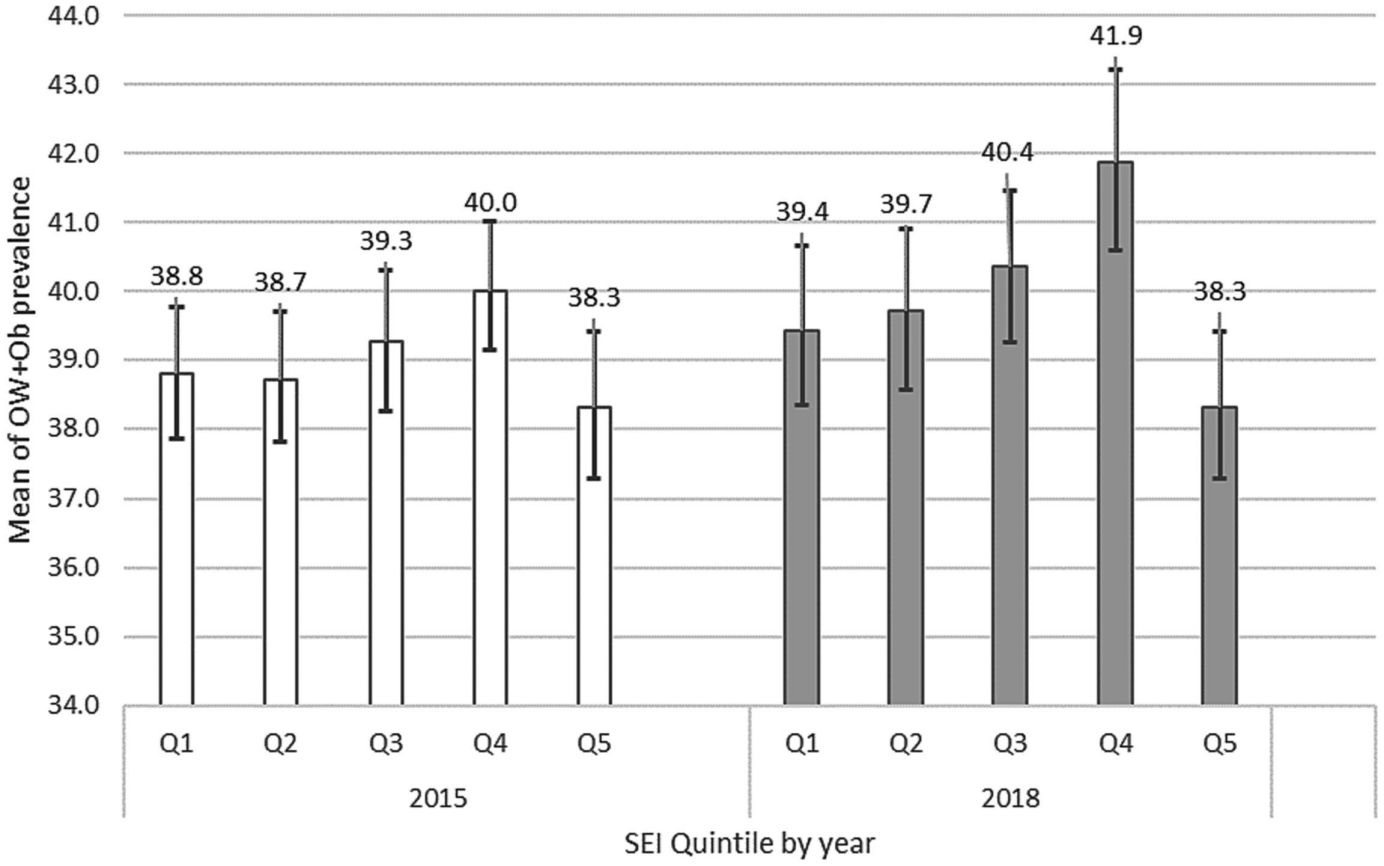
Mean and 95%CI of OW + Ob prevalence in schools in Monterrey Mexico Metropolitan Area by Social Economic Index (SEI) quintile 2015–2018.

To assess whether variations in socioeconomic status alone could account for differences in OW + Ob prevalence among schools, a bootstrap analysis of mean prevalence and 95% confidence intervals (CI) was conducted based on Socioeconomic Index (SEI) quintiles. The results, depicted in Figure 1, indicate that although Q4 consistently exhibited a higher prevalence in both years and the overall prevalence increased from the first to the second year of the study, no statistically significant differences were evident between the quintiles in 2015 and 2018 (with overlapping CIs).

### 3.3 Convenience store behavior between 2015 and 2018

Convenience stores registered in the National Directory of Economic Units (DNUE) in the geographical area of the study grew from 1,394 to 1979 in the 2015–18 period, which corresponds to a 42% relative increase in the number of stores in the cited period. Another way to describe this growth is that from 2015 to 2018, a new store was opened every 2.5 days in the Monterrey Metropolitan Area.

### 3.4 The number of stores and their impact on OW + Ob

Table 2 shows the mean and SD of the number of convenience stores in the OW + Ob quintile for the 2 years of the present study. The first aspect that is clear is that regardless of the store’s density, in Q4 and Q5, OW + Ob increased between 2015 and 2018, showing a greater lower limit value in the most recent period (40.6 to 41.0 and 44.8 to 46.6, respectively). In other words, in the same proportion of the Q4 and Q5 quintiles, the actual prevalence is greater. Another interesting finding is that, between 2015 and 2018, in all quintiles and for both radii, the mean number of stores increased. If we consider that schools are spatially fixed, this necessarily indicates that a significant number of stores opened in the school’s proximity during this time. A greater increment can be seen within the 400 m radius for Q1 schools, which increased from 0.82 schools to 1.27 (this corresponds to a 54% relative increment).

**Table 2:** Mean and standard deviation of the number of convenience stores around the elementary schools within 400 m and 800 m radii by OW + Ob quintile and year in Monterrey Mexico Metropolitan Area 2015–2018.

2015										
	Q1		Q2		Q3		Q4		Q5	
	*N* = 310 OW + Ob % (< 32.3)		*N* = 310 OW + Ob % (≥32.3 – <36.9)		*N* = 311 OW + Ob % (≥36.9 – <40.6)		*N* = 311 OW + Ob % (≥40.6– <44.8)		*N* = 310 OW + Ob % (≥44.8)	
	Mean	S.D.	Mean	S.D.	Mean	S.D.	Mean	S.D.	Mean	S.D.
400 m radius	0.82	1.02	1.02	1.28	1.14	1.44	1.15	1.79	1.27	1.53
800 m radius	3.1	3.08	3.7	3.41	4.27	4.14	4.46	4.8	5.16	4.55
2018										
	Q1		Q2		Q3		Q4		Q5	
	*N* = **310** **OW +** Ob % (< 32.3)		*N* = 311 OW +Ob % (≥32.3–<36.9)		*N* = 310 OOW + Ob % (≥36.9–<41.0)		*N* = 311 OW + Ob % (≥41.0–<46.6)		*N* = 310 OW + Ob % (≥46.6)	
	Mean	S.D.	Mean	S.D.	Mean	S.D.	Mean	S.D.	Mean	S.D.
400 m radius	1.27	1.22	1.4	1.54	1.59	1.87	1.64	1.47	1.96	2.36
800 m radius	4.34	3.12	5.34	4.63	6.24	5.64	6.32	3.9	7.06	6.34

**Figure 2:**
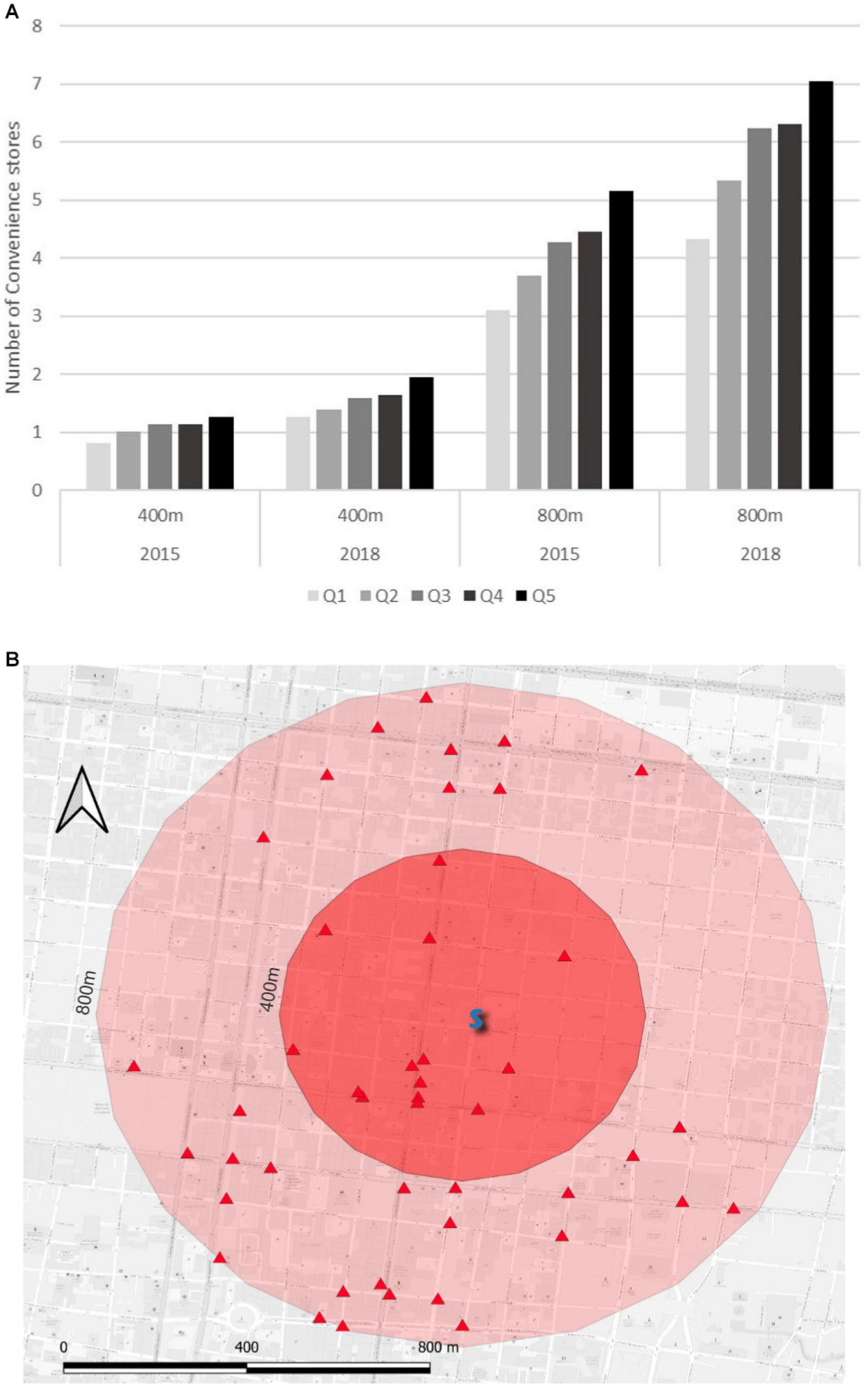
**(A)** Mean of the number of convenience stores around the elementary schools within 400 m and 800 m radii by OW + Ob quintile and year in Monterrey Mexico Metropolitan Area 2015–2018. **(B)** Extract of the actual data showing a school (S) surrounded by 14 and 46 convenience stores (Red triangles) in radii of 400 m and 800 m, respectively.

The relationship between the OW + Ob quintile and the number of stores in the two radii is shown in Figure 2A which graphically shows two trends: the OW + Ob quintile increases with the number of stores and, as shown in Table 2, the OW + Ob quintile and the store number increased between 2015 and 2018. The same Figure 2B shows an example of a school with 14 convenience stores within its 400 m radius and the remarkably high number of 46 within its 800 m radius.

The results show that, in all cases, a greater number of stores were present in the higher OW + Ob quintiles; moreover, a consistent trend was found as the quintile increased (perfect ladder view in Figure 2). It was quite concerning that by 2018, the children of the more prevalent OW + Ob schools could reach more than seven stores by walking for 10 min and at least two stores by walking for only 5 minutes. Another finding of this analysis was that over 3 years, all schools, regardless of their OW + Ob status, faced an increase in the density of convenience stores on their periphery.

### 3.5 Distance to the nearest convenience store and its impact on OW + Ob

**Figure 3:**
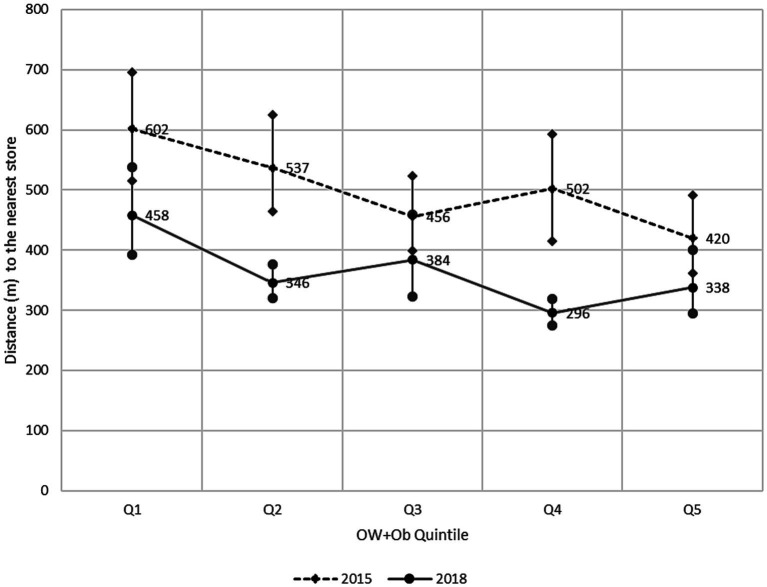
Mean Euclidean distance (m) and 95%CI between schools and the nearest convenience store by OW + Ob quintile, Monterrey Mexico Metropolitan Area 2015–2018.

Regarding the influence of the proximity of convenience stores and its effect on OW + Ob prevalence, Figure 3 shows a decreasing trend of the mean distance to the nearest store as the prevalence increases for 2015 and 2018; in other words, the schools in which the OW + Ob is greater have, on average, a convenience store nearer than those with lower prevalences (182 m for 2015 and 120 m for 2018). It is also clear that between 2015 and 2018, for all schools, regardless of the OW + Ob level, stores were actually nearer.

In this sense, we also found that by 2018, the average distance for the schools with the highest prevalence was only 338 m, but even for those with a lower prevalence, the average distance was 458 m, which indicates that in all cases, convenience stores and their food were near the schools. In this analysis, we confirmed the increase in the number of convenience store trends found in the density computations, which can be seen in Figure 3, in which the line of 2018 is well below the 2015 one, indicating that convenience stores and junk food offerings became nearer and thereby easier and faster to access during this period.

**Figure 4:**
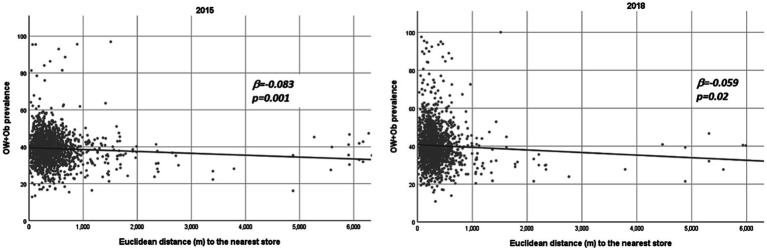
Scatterplot, linear fit, a negative beta value of *p* of the distance to the nearest convenience store (trimmed to 6 km) vs. OW + Ob prevalence in Monterrey Mexico Metropolitan Area 2015–2018.

Another way to visualize the relationship between OW + Ob and distance to the nearest store is by plotting the schools as data hotspots using the school-store distance as the X-axis and the OW + Ob prevalence as the Y-axis and making a linear regression of the dataset, which can be seen for both years in Figure 4, where a negative slope of the linear fit can be seen and is confirmed by the negative β (beta) value, indicating that a trend for a lower prevalence of OW + Ob occurs as the schools have their nearest school at a greater distance. When viewing the same data not aggregated in quintiles but as continuous hotspots (Figure 4), one can confirm the cited behavior by inspecting the plot and linear regression negative coefficients that, again, not only confirm but also prove with statistical significance the inverse relationship between the distance to the nearest store and OW + Ob prevalence.

### 3.6 Cartographical visualization of OW + Ob and store density

**Figure 5:**
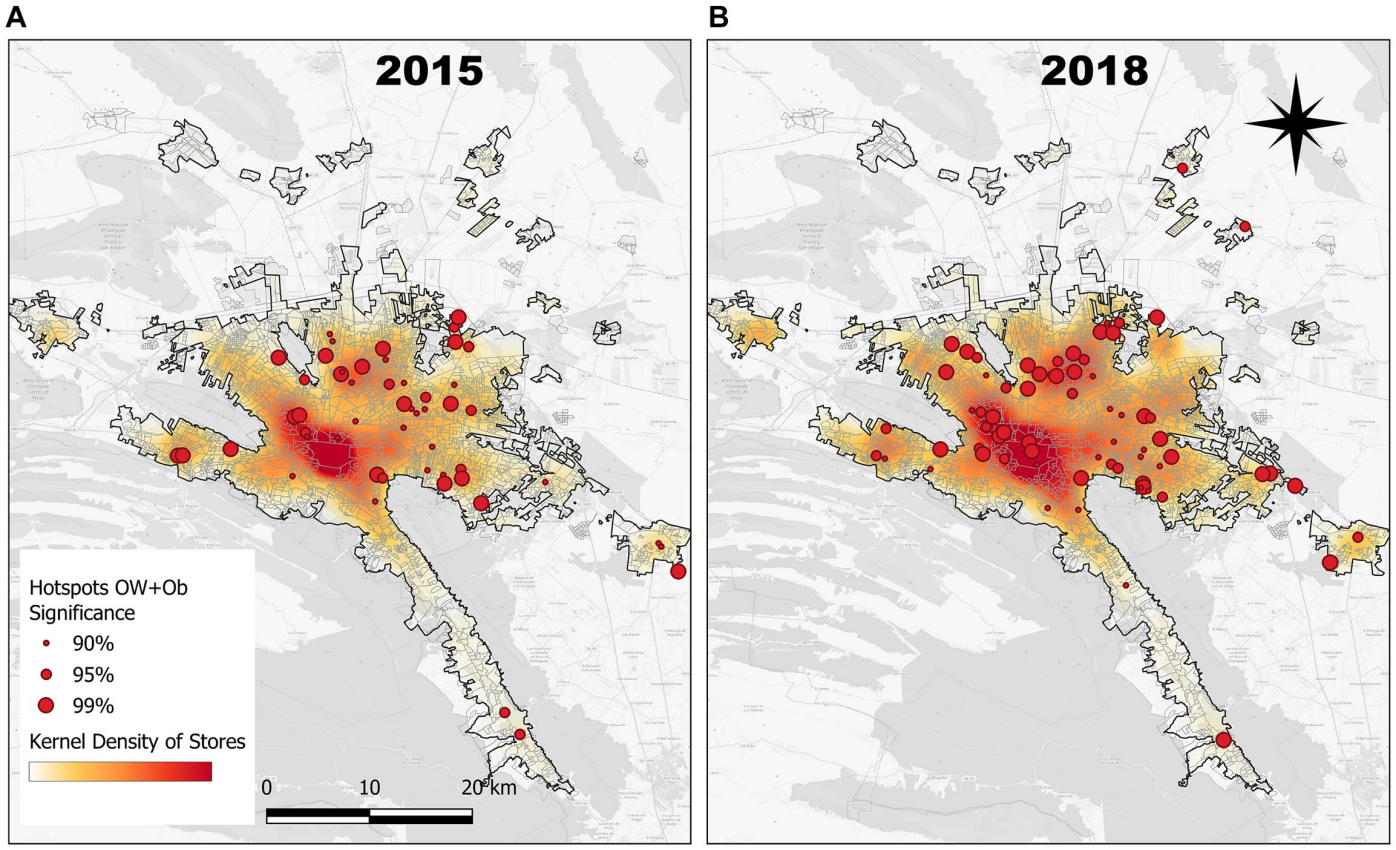
Getis-Ord Gi* Hotspots and significance level of OW + Ob in elementary school children in comparison to kernel density of convenience stores in Monterrey Metropolitan Area, Mexico in **(A)** 2015 and **(B)** 2018.

Figure 5 shows two maps of the Metropolitan Area of Monterrey for 2015 (Figure 5A) and 2018 (Figure 5B), in which a raster layer in the background corresponding to the kernel density of convenience stores is presented as a white–red color ramp, where the more intense red color represents areas with a high density of stores. Another information layer presented as size-scaled hotspots shows those schools that were detected by the *Getis*-Ord Gi*algorithm as OW + Ob *hotspots*; in other words, the schools that presented a much higher prevalence of OW + Ob when compared to their neighbors. Point size indicates the confidence level of the categorization as a *hotspot* (90, 95, or 99%).

Looking at the maps side by side, it is clear that (regardless of the OW + Ob component) the store density grew from 2015 to 2018; it can be seen that (b) shows a more intense red background in the central area of the city and a slight increase in the periphery when compared to (a), which is a visual confirmation of the findings presented in Table 2. Regarding the hotspots, in (a), there are 63, whereas in (b), this cipher increased to 91, being an important proportion of such hotspots of 95–99% significance. It is also important to point out that in the central area of the city where the store density is greater, an important number of OW + Ob hotspots appeared over 3 years. Overall, the maps displayed a general concordance between the store’s denser areas and the OW + Ob hotspots for both years, but this was more evident for the most recent period.

The store density and OW + Ob *Getis*-Ord Gi* Hotspots maps allow not only the visualization of the spatial distribution of the two variables but also the changes over time. Focusing on store density, it is clear that they are more abundant in the center of the metropolitan area and that they increased in number between 2015 and 2018 in the same center and also toward the periphery. The maps also show that in the areas of higher store density in the center and north, there are more schools with high OW + Ob, and the presence and significance levels of the hotspots increased between 2015 and 2018. It is also shown, in a more subtle way, that some new hotspots appeared in the periphery and some had an increased significance level.

## 4 Discussion and conclusions

Monterrey Mexico is a metropolitan area that belongs to the Nuevo León State, where, in the 2022 National Continuous Nutrition Survey, there was an OW + Ob prevalence of 34.2% (39), whereas the National was 37.1% (4). These data are strongly consistent with the prevalences found by us in 2015 and 2018 (Table 1). This region, especially the city, is highly industrialized, and its cultural and social patterns are strongly influenced by its proximity to the United States. One of the results of such an influence can be found in current eating behaviors, which have shifted from traditional food to fast food and energy-dense food (40). This is also where the main chain of convenience stores (OXXO) opened its first business more than 46 years ago (41, 42). This metropolitan area is also the second most populated in the country (43), and all the previous factors make this place suitable for conducting the analyses of the present work.

It is well known that OW + Ob is a complex phenomenon that cannot be reduced to one or only a few factors. However, one of the main questions that could be addressed in this study is whether the stores themselves are the influencing factors for OW + Ob development or whether the mere presence of the stores in some places reflects the intrinsic economic characteristics of the regions inside the metropolitan area, and whether such characteristics are what finally determine or at least have an impact on OW + Ob. To control this issue, as the first step of the study, we calculated the distribution of OW + Ob using an SEI, finding no significative differences between the groups; this led us to discard the idea that inside the Monterrey Metropolitan area, the problem of OW + Ob in the children was determined mainly by socio-economic characteristics.

Approaching the research question from a spatial perspective, the first influencing factor from the stores that were studied was their density around the schools within 400 m and 800 m radii, corresponding to walking times of 5 and 10 min, respectively (32). This was important because we wanted to determine the number of junk/fast food offering hotspots available within those two radii around the schools. In other words, we considered these variables as a measure of the magnitude of this kind of food availability for all children who attended schools.

The other spatial property that we studied in the school–store interactions was nearness, which can be interpreted as the ease of access to junk/fast food by the children. These results are consistent with the density. The use of Euclidean distance constitutes the first approach; however, more sophisticated methods, such as distances over street networks (service areas) could be used in future research.

When viewing both maps side-by-side, it seems that the store presence and the obesity hotspots spread over time, increasing in the center and expanding these phenomena to the edges of the Metropolitan Area. Again, these analyses correspond to the first approach but are susceptible to being enhanced in many ways: updating the data (schools and stores) to more recent sets to confirm the cartographic findings, performing other analyses (i.e., map algebra) using density rasters, and perhaps replicating these same methods in other regions of the country. Although we cannot state that the store density, distance, and location are directly responsible for the OW + Ob condition of children attending school, we found interesting and consistent facts and trends that may point toward this. Although there are few spatial analyses of this issue in Mexico, the obtained results are consistent with those found by Zavala et al. in 2021 (44), but in the case of the present work, on a much larger scale (metropolitan area) and over a lapse of time. Furthermore, these results are consistent with numerous studies on urban areas in developed countries (45, 46).

According to a systematic review by Matsusaki et al. (47), a positive association exists between the nearness of fast food selling hotspots to schools and OW + Ob prevalence in children from Latin, Anglo-Saxon, and Afro-American ethnic groups from different continents, which was observed in all school degrees, but the authors remarked that this could be more important in younger individuals.

Hughey and collaborators (48) proposed a kernel density estimator methodology for an adolescent population to group the obesogenic environment components in the proximity of neighborhoods, such as processed food selling hotspots and fast food restaurants, and, on the other hand, positive elements such as parks and green/recreative areas used for physical activity. The authors recognize all of these places as relevant to the research because of the large amount of time spent by the individuals there.

In a similar study, Buszkiewicz et al. (49) established a relationship between the obesogenic environment and the presence of OW + Ob according to the place of residence. Their main findings agree with our work by showing that at smaller distances between households and junk food selling hotspots, OW + Ob prevalence increases. These results reinforce the idea that variables related to the food environment have an impact on weight gain.

The strengths of this study include its use of a large amount of data (census) on a homogeneous population (school-attending children), which results in robust OW + Ob ciphers, along with consistent and complete spatial information about convenience stores. This, in the context of a metropolitan area that does not have the enormous economic/social inequities present in other regions, allowed for the removal of some of the “noise” and facilitated a focus on the impact of the stores on OW + Ob. On the other hand, the study’s weakness consists of the multifactorial complexity of the problem, which cannot be reduced only to junk food consumption and, thus, the stores’ geographical locations. Another limitation could lie in the simplicity of some of the methods used (buffers and Euclidean distance), which could certainly be improved in future research. Despite the fact that it is not possible to confirm that all of the junk food and sweetened beverages come from convenience stores, it remains a hard fact that the number of such businesses has grown in recent years, reducing the distance of access to ultra-processed foods. This rapid spread of stores could not be explained without having significant sales, and in contrast to the supermarkets that are centralized meeting hotspots, these stores’ strategies consist of “getting nearer” to the customers and readily offering the products.

In conclusion, investigating the relationship between convenience stores and their role in obesity in school-aged children, which is a growing global public health concern, requires an understanding of the potential role of convenience stores in contributing to this problem to support interventions and policies aimed at mitigating its impact, along with the use of spatial analysis, which provides valuable information for public health interventions and urban planning and aids in tailoring interventions to specific geographic areas and formulating policy to promote healthier environments for children to grow and develop in. Considering this, it is important to conduct more research on this topic in Mexico using more recent datasets and more sophisticated methods to identify the precise role and possible negative impacts of convenience stores on the health of the population. It is necessary to promote guidelines that restrict the availability of junk food and sugar-sweetened beverages in schools.

Public policies aimed at effectively coping with this problem must include strategies for dealing with obesogenic environments. This implies limiting the number of convenience stores around schools and, at the same time, promoting the availability of healthy foods.

## Data availability statement

The raw data supporting the conclusions of this article will be made available by the authors, without undue reservation.

## Author contributions

MÁ: Writing – original draft, Investigation, Formal analysis, Conceptualization. TS: Writing – original draft, Supervision, Formal analysis, Conceptualization, Writing – review & editing. MD: Methodology, Data curation, Writing – review & editing. AC: Writing – review & editing, Project administration, Investigation, Conceptualization. AÁ: Writing – review & editing, Project administration, Investigation.
